# Supplementary material for: Multi-site clonality analysis uncovers pervasive heterogeneity across melanoma metastases
Source: Nat Commun. 2020 Aug 27;11:4306. doi: 10.1038/s41467-020-18060-0 (PMC7453196; doi:10.1038/s41467-020-18060-0)
Supplement: Supplementary file 1 — Supplementary Information [file 41467_2020_18060_MOESM1_ESM.pdf]

Multi-site clonality analysis uncovers pervasive heterogeneity across melanoma metastases.

Rabbie *et al.* 2020

Supplementary Information

Supplementary Figure 1

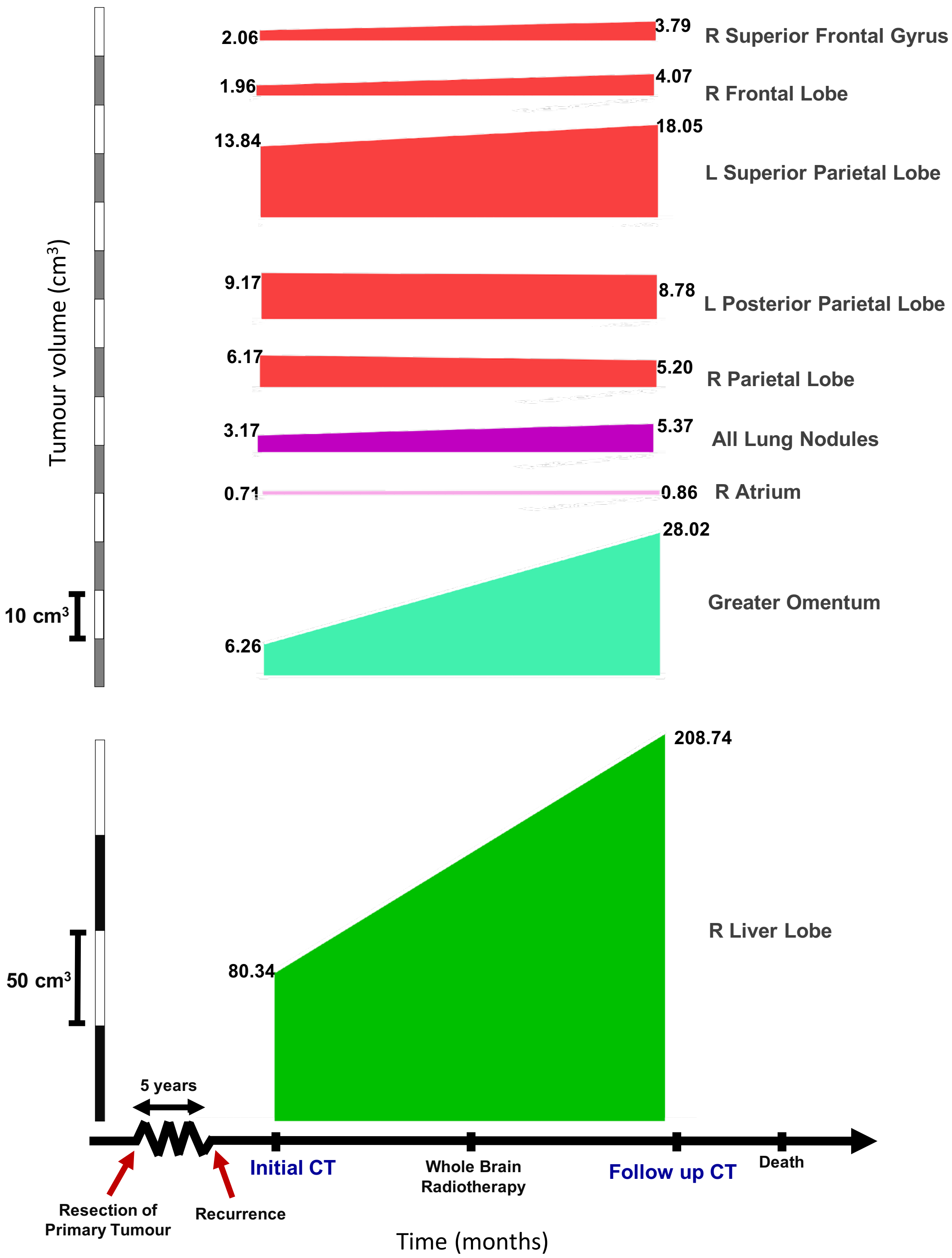

Supplementary Fig 1. Representative clinical timeline of the index autopsy case, demonstrating rapid progression from the first appearance of metastatic disease. The volume changes of target lesions between interval CTs performed 5 weeks apart are shown, with the follow-up imaging taken two weeks after the completion of whole-brain radiotherapy. This showed only minimal intracranial, but extensive extracranial disease progression.

Supplementary Figure 2

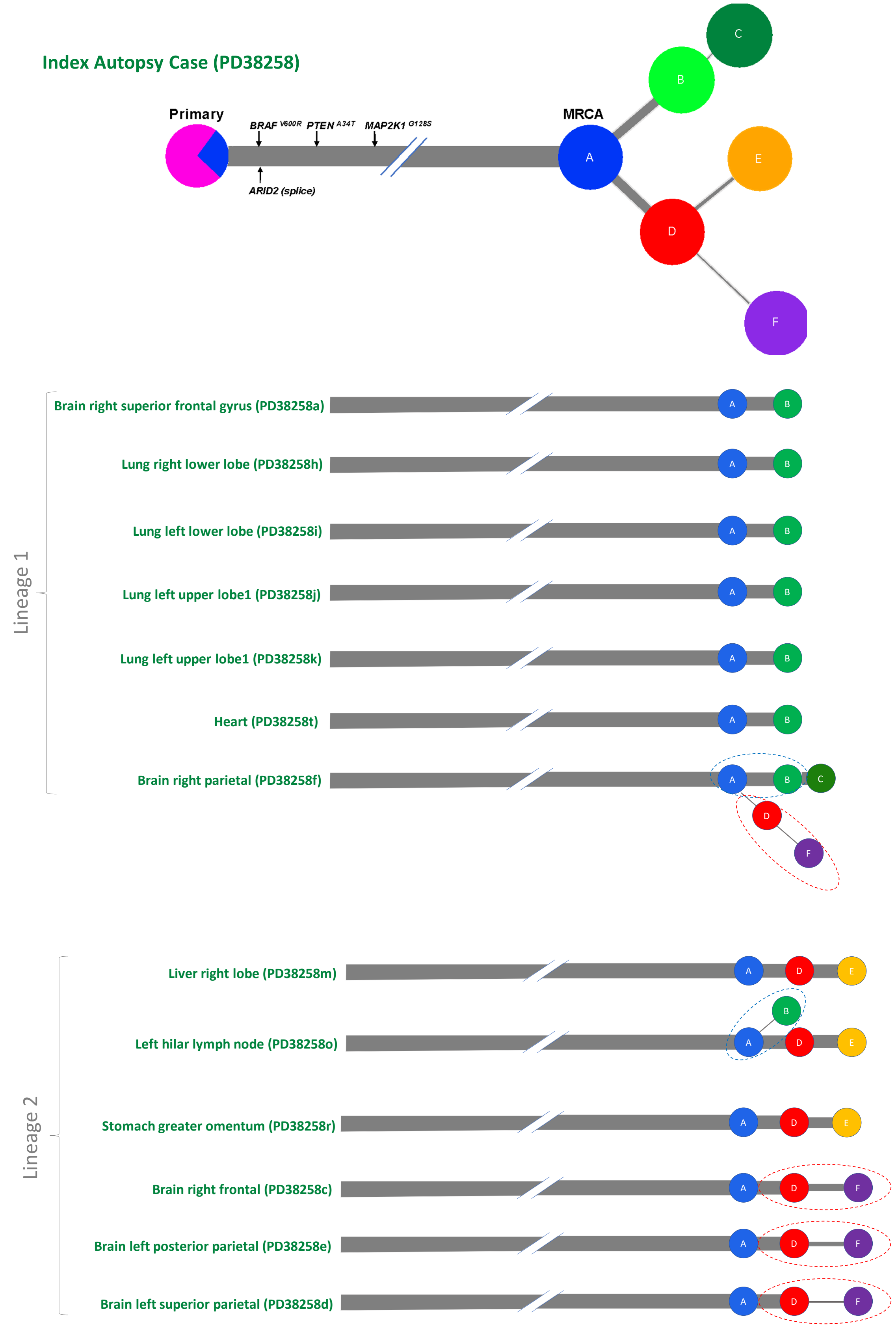

Supplementary Fig 2. Sample-level phylogenetic tree for the index autopsy case. Each tree represents a subtree of the overall phylogenetic tree (**Fig. 2D**) including just those subclones seen within that particular sample. However in doing this we were able to segregate the samples based on their respective clonal lineages. We observed two clear lineages, representing distinct waves of metastatic seeding depicted here as the lineage 1 and 2 emanating from clusters B (light green) and clusters D (red) respectively. Dotted ovals represent evidence for polyclonal seeding. Subclones within each oval are found with differing CCFs in 2 or more samples.

Supplementary Figure 3

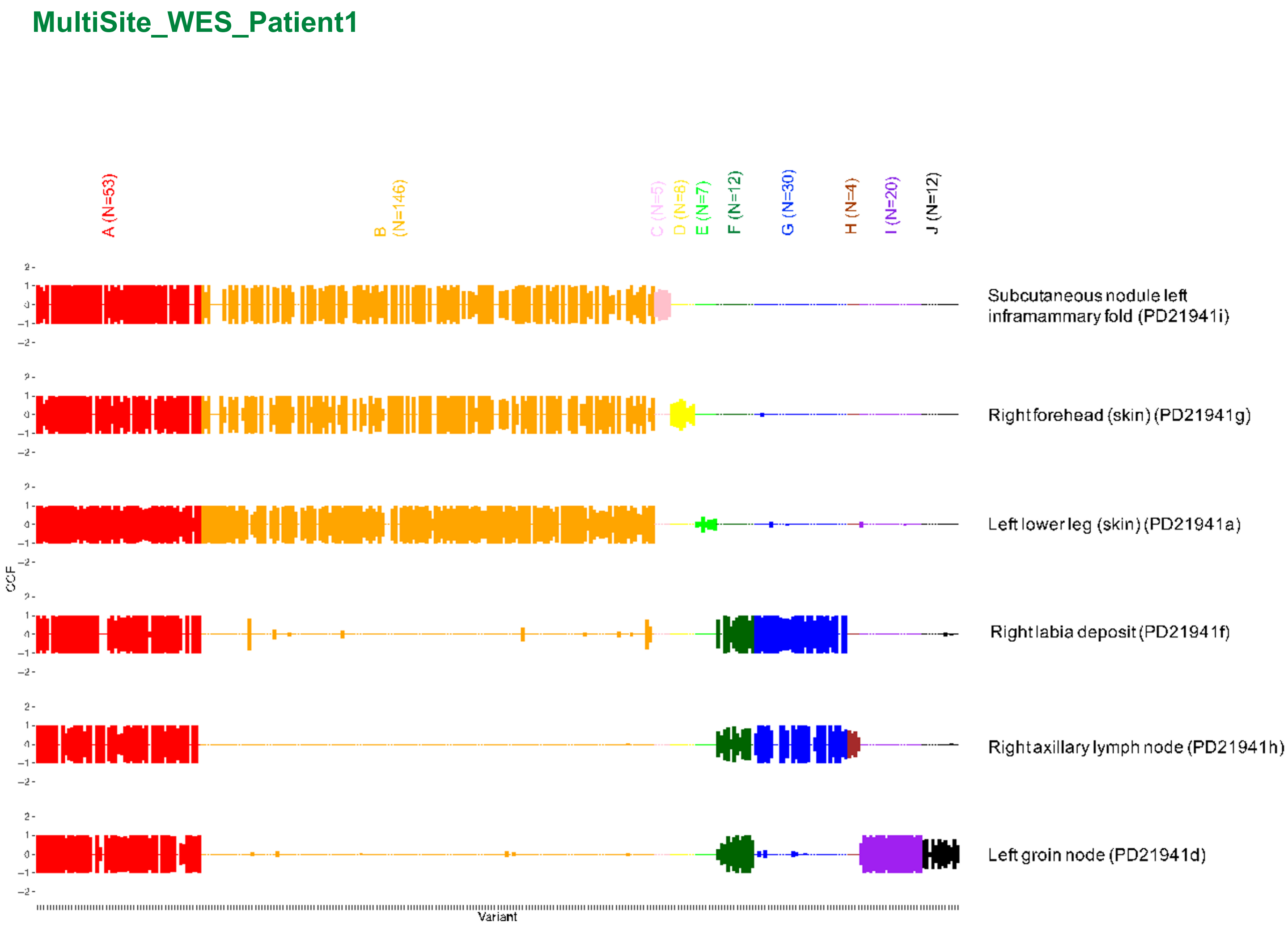

Supplementary Fig 3. CCF distribution plot for whole-exome sequenced patient MultiSite_WES_Patient1. Rows reflect samples and columns reflect alphabetically and colour-coded mutation clusters (number of SNVs within each cluster is indicated at the top). This shows that clusters B (yellow) and F (green) were clonal in mutually exclusive samples and represent mutually exclusive clonal phylogenies at the first bifurcation of the phylogenetic tree.

Supplementary Figure 4

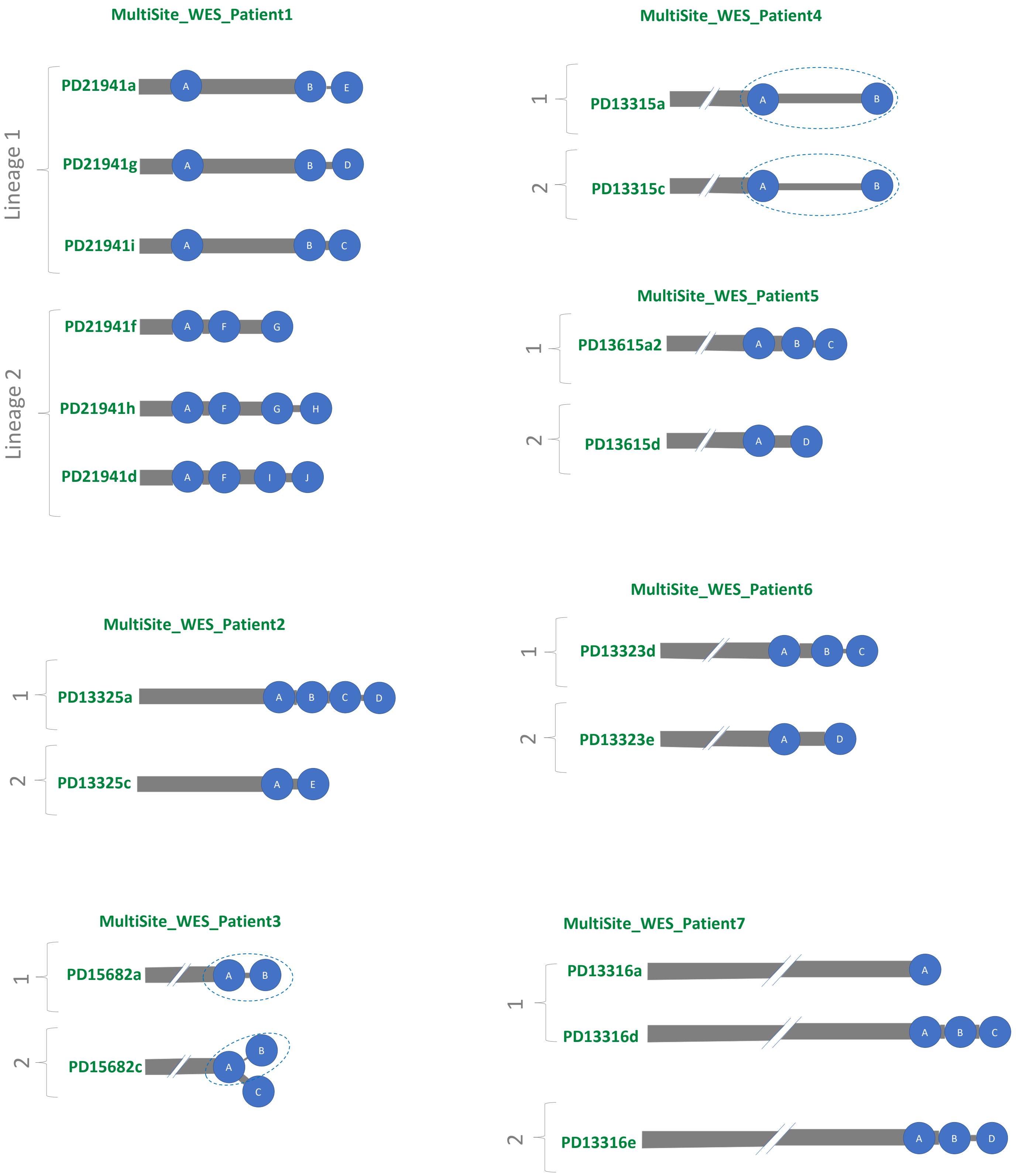

Supplementary Fig 4. Sample-level phylogenetic tree for multi-site whole-exome sequenced cases. The respective branched lineages are depicted for each patient. Dotted ovals represent evidence for polyclonal seeding. Subclones within each oval are found with differing CCFs in 2 or more samples. Only two patients (MultiSite_WES_Patient3 and MultiSite_WES_Patient4) displayed polyclonal seeding.

Supplementary Figure 5

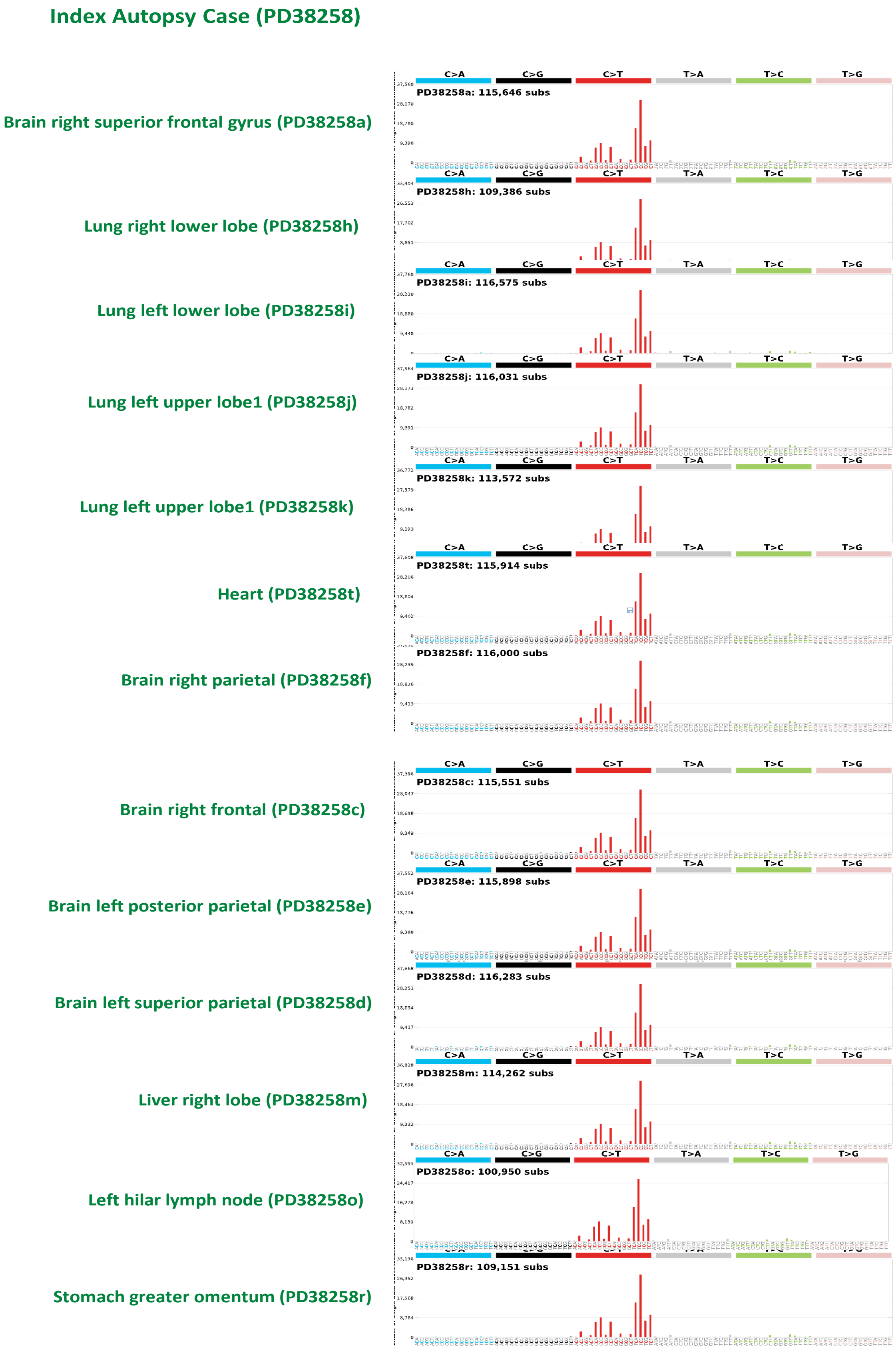

Supplementary Fig 5. Mutational signatures for all SNVs from the index autopsy case. Shows the mutational profile using the conventional 96 mutation type classification as described by Alexandrov and colleagues[31,49]. This classification is based on the six substitution subtypes: C>A, C>G, C>T, T>A, T>C, and T>G. Further, each of the substitutions is examined by incorporating information on the bases immediately 5' and 3' to each mutated base generating 96 possible mutation types. Here we show the signature profiles including all SNVs from all 13 WGS metastases which as expected, were dominated by signature 7.

Supplementary Figure 6

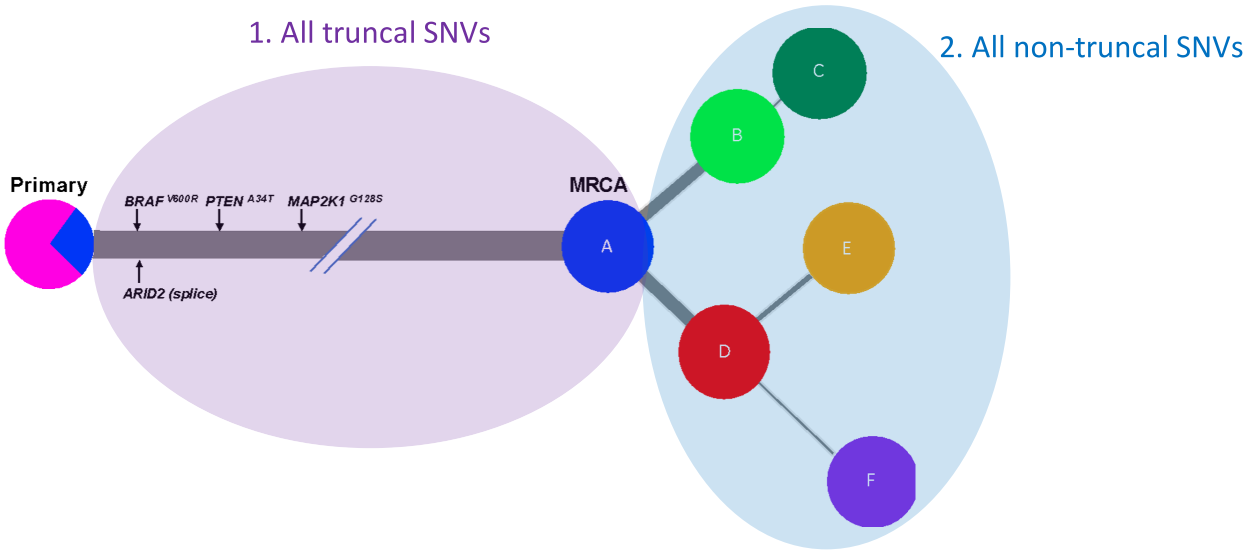

A

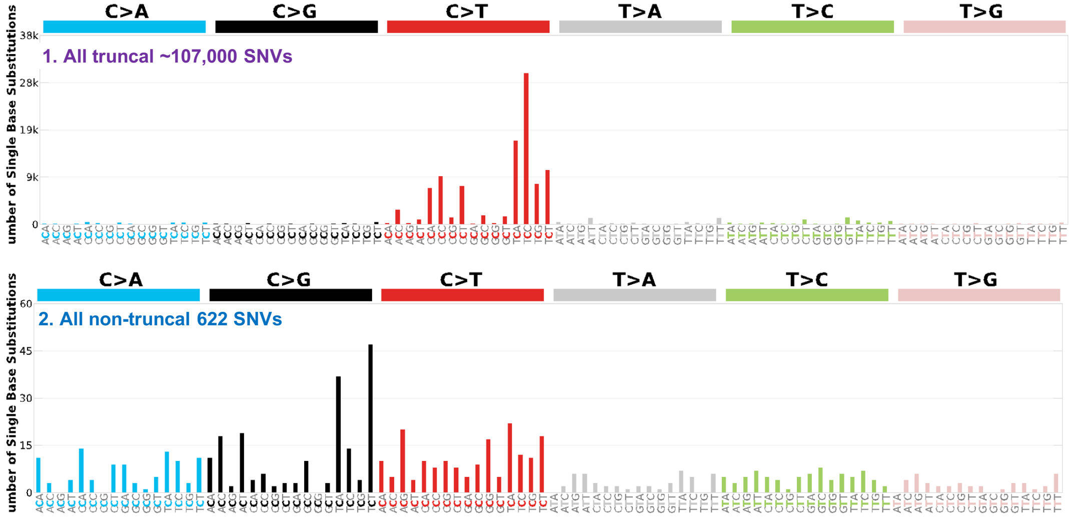

B

Cluster Groups	Global NMF Signatures	Similarity
1. All truncal SNVs	Signature Subs-01 (0.90%)<br>Signature Subs-05 (9.01%)<br>Signature Subs-07 (90.09%)	1.00
2. All non-truncal SNVs	Signature Subs-01 (9.65%)<br>Signature Subs-02 (3.22%)<br>Signature Subs-05 (5.95%)<br>Signature Subs-13 (16.08%)<br>Signature Subs-40 (65.11%)	0.95

Supplementary Fig 6. Mutational signatures from the index autopsy case. A) Here we show the mutational profiles including; all truncal SNVs in the tree (n~107,000 SNVs) and all non-truncal SNVs (from the six mutation clusters B-F) in the tree (n=622 SNVs). B) The Global NMF signatures shown represent the 'best fit' signatures across all SNVs and the individual percentages for each signature is the proportion which that signature represents. The cosine similarity reports how closely these signatures together mirror the context of all SNVs within that cluster group. As expected, the mutational signature for all truncal SNVs is dominated by signature 7 (90% of SNVs are represented by this signature) whilst this is entirely absent from the non-truncal clones, which are represented by the APOBEC mutational signatures (2 & 13). The non-highlighted signatures (1, 5 and 40) shown in black represent relatively featureless ("flat") oncogenic signatures found in most cancer types and do-not as yet define any distinguishing biological processes[49].

Supplementary Figure 7

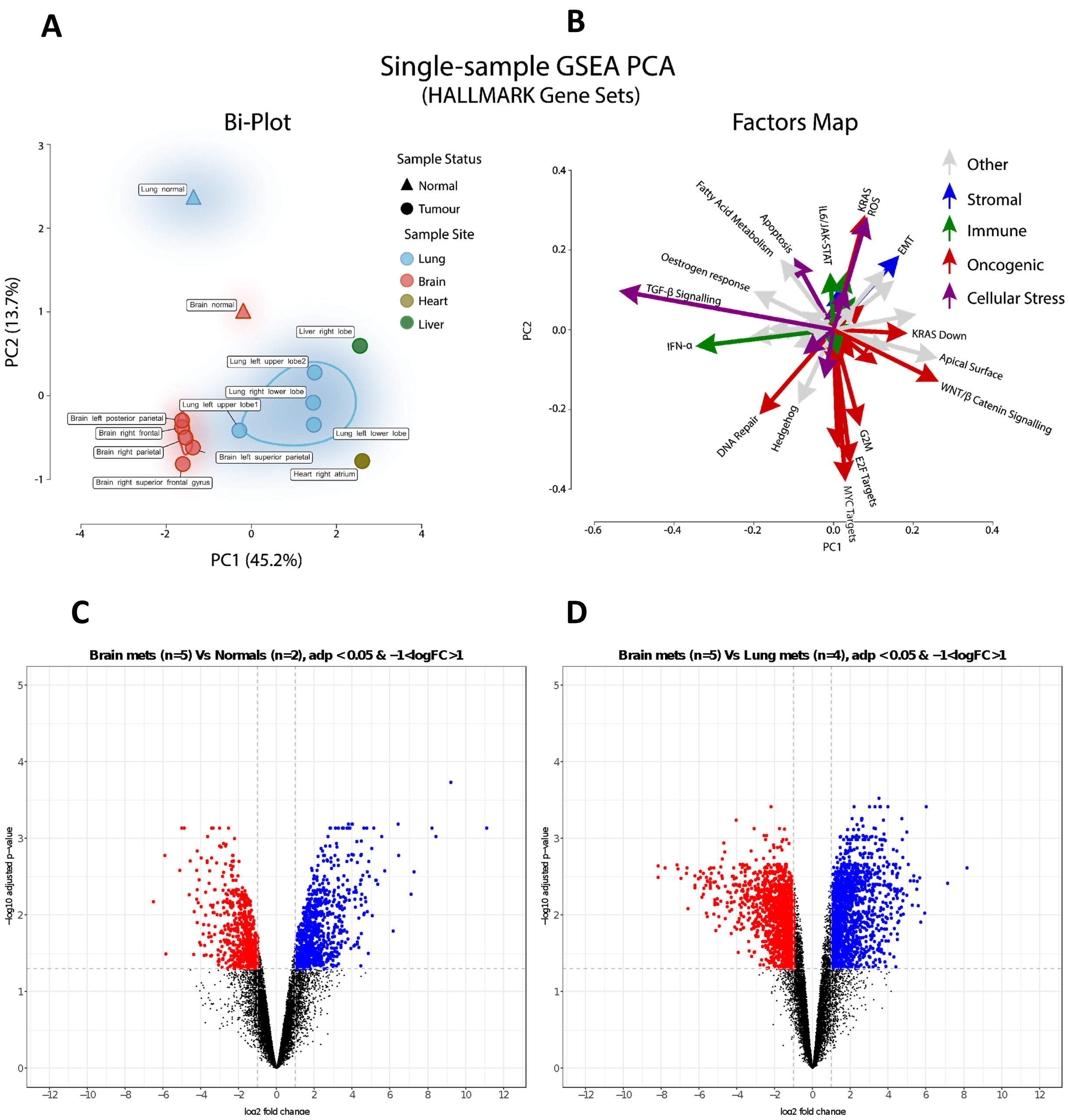

Supplementary Fig 7. Differential expression and principal component analyses from the index autopsy case. A) Principal component analysis (PCA) of single sample gene-set enrichment (ssGSEA) using the hallmark gene sets (see **Methods**)[51]. A similar pattern of regional separation (represented in **Fig. 4A**) was observed between the brain and lung metastases, which again are separated from the corresponding patient-matched normal organ control samples. Samples are circled using a kernel density estimation. B) Principal component feature loadings (magnitude and direction) of (A) are shown in the variables factor map. Each co-ordinate in (B) reflects the correlation coefficient of the biological process to principal components 1 (x-axis) and 2 (y-axis) from (A). Vectors are coloured according to the major biological classification of Hallmark gene sets. This revealed that PC2 (on the y-axis), explaining the variation between the tumour and normal samples (represented by circles and triangles in (A) respectively), is primarily represented by the up-regulation of oncogenic processes highlighted with a red arrow pointing (downwards) towards the tumour samples. C) Volcano plot of the genes differentially expressed between the brain metastases (n=5) versus the patient-matched normal tissue (one sample from brain and lung respectively, n=2). Each dot represents one gene, dots above the dotted line are considered statistically significant (FDR-adjusted p-value <0.005 calculated in limma, see methods) and are shaded according to fold-change cut-offs (log fold-change < -1 coloured in red, and log fold-change >1 in blue). D) Volcano plot of the genes differentially expressed between the brain (n=5) versus lung metastases (n=4). Dots represent genes, coloured in the same format as (C). FDR-corrected p-values are calculated in limma, see methods.

Supplementary Figure 8

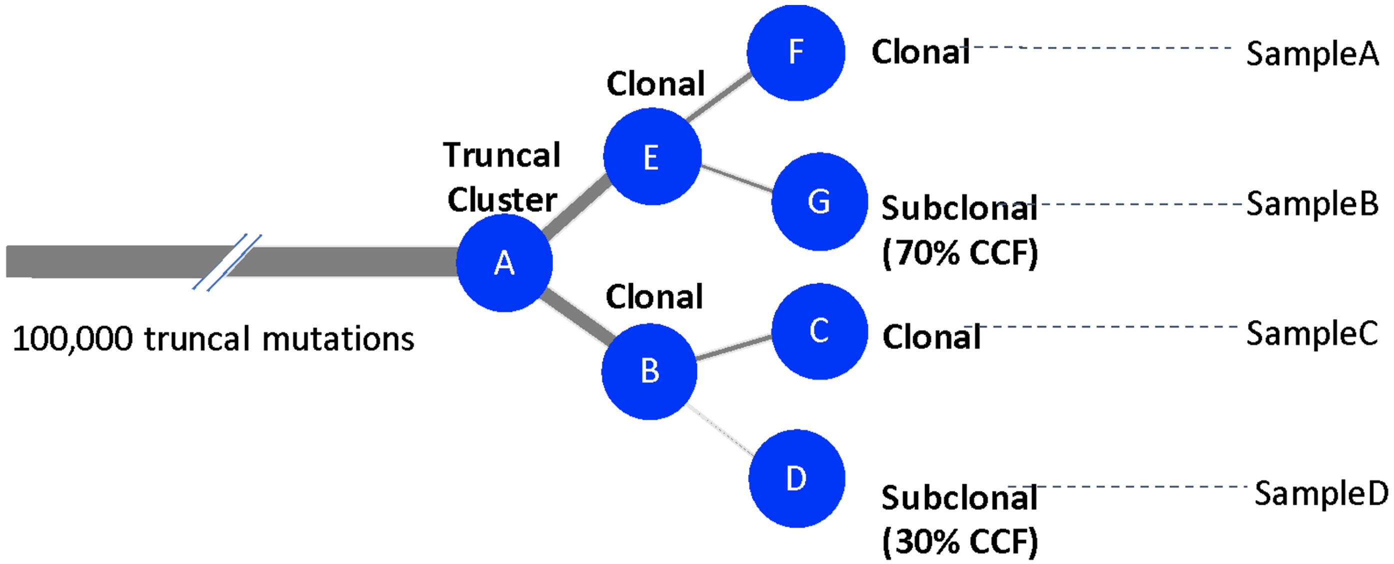

Supplementary Fig 8. Simulations of phylogenetic tree reconstructions from the index autopsy case with variable subclonal heterogeneity.

Supplementary Table 1. Summary of key clinical and sample details relating to the index autopsy case. The raw sequencing files and variant/CNV calls are all deposited in data availability.

Patient	MelResist_PatientID	Sex	Melanoma subtype	Date of initial melanoma diagnosis	TSTAGE (at initial melanoma diagnosis)	NSTAGE (at initial melanoma diagnosis)	MSTAGE (at initial melanoma diagnosis)	Date of trial registration	Number of lines of therapy	Treatment name	Treatment start date	Treatment end date	WGS_SampleID	AffySampleID	Tumour/Normal	Sample_resection_date	Sample_type	Site	Depth	Purity_Estimate
Index_autopsy_case	01_123	Male	Cutaneous	07/2012	T2a	N0	M0	10/2017	1	Whole-brain RT	07/2017	'07/2017	PD38258a	PR38258a	Tumour	09/2017	Brain	Brain_right_superior_frontal_gyrus	33.5134	0.94
Index_autopsy_case	01_123	Male	Cutaneous										PD38258b	NA	Normal	NA	Buffy coat	Germline_buffy_coat	37.9218	NA
Index_autopsy_case	01_123	Male	Cutaneous										PD38258c	PR38258c	Tumour	09/2017	Brain	Brain_right_frontal	36.21725	0.728
Index_autopsy_case	01_123	Male	Cutaneous										PD38258d	PR38258d	Tumour	09/2017	Brain	Brain_left_superior_parietal	37.81692	0.856
Index_autopsy_case	01_123	Male	Cutaneous										PD38258e	PR38258e	Tumour	09/2017	Brain	Brain_left_posterior_parietal	36.1521	0.821
Index_autopsy_case	01_123	Male	Cutaneous										PD38258f	PR38258f	Tumour	09/2017	Brain	Brain_right_parietal	41.0643	0.857
Index_autopsy_case	01_123	Male	Cutaneous										PD38258h	PR38258h	Tumour	09/2017	Lung	Lung_right_lower_lobe	43.87209	0.511
Index_autopsy_case	01_123	Male	Cutaneous										PD38258i	PR38258i	Tumour	09/2017	Lung	Lung_left_lower_lobe	41.63439	0.89
Index_autopsy_case	01_123	Male	Cutaneous										PD38258j	PR38258j	Tumour	09/2017	Lung	Lung_left_upper_lobe1	33.03544	0.883
Index_autopsy_case	01_123	Male	Cutaneous										PD38258k	PR38258k	Tumour	09/2017	Lung	Lung_left_upper_lobe2	39.93535	0.709
Index_autopsy_case	01_123	Male	Cutaneous										PD38258m	PR38258m	Tumour	09/2017	Liver	Liver_right_lobe	36.84432	0.884
Index_autopsy_case	01_123	Male	Cutaneous										PD38258o	NA	Tumour	09/2017	L hilar lymph node	LN_left_hilar	39.42053	0.422
Index_autopsy_case	01_123	Male	Cutaneous										PD38258r	NA	Tumour	09/2017	Stomach	Stomach_greater_omentum	38.36801	0.586
Index_autopsy_case	01_123	Male	Cutaneous										PD38258t	PR38258t	Tumour	09/2017	Heart	Heart_right_atrium	40.30025	0.823
Index_autopsy_case	01_123	Male	Cutaneous										PD38258u	NA	Tumour	07/2012	Primary	Primary_anterior_chest_wall1	NA	NA
Index_autopsy_case	01_123	Male	Cutaneous										PD38258v	NA	Tumour	07/2012	Primary	Primary_anterior_chest_wall2	NA	NA

Supplementary Table 2. Summary of key clinical and sample details relating to the multisite whole-exome sequenced cases. The raw sequencing files and variant/CNV calls are all deposited in data availability.

Patient	MelResist_PatientID	Sex	Melanoma subtype	Date of initial melanoma diagnosis	TSTAGE (at initial melanoma diagnosis)	NSTAGE (at initial melanoma diagnosis)	MSTAGE (at initial melanoma diagnosis)	Date of trial registration	Number of lines of therapy	Treatment name	Treatment start date	Treatment end date	WES_SampleID	Tumour/Normal	Sample_resection_date	Sample_type	Site	Target_Region_Coverage_AfterPCRDupRemoval
MultiSite_WES_Patient1	MR01_034	Female	Acral	12/2003	**T4b**	N0	M0	03/2014	2	Roche/RAF/MEK Inhibitor	07/2010	09/2011	PD21941i	Tumour	06/2015	Distant skin/subcutanoues	Subcutaneous nodule left inframammary fold	34.5
MultiSite_WES_Patient1	MR01_034	Female	Acral							Ipilimumab	10/2011	12/2011	PD21941f	Tumour	04/2015	Distant skin/subcutanoues	Right labia deposit	37.4
MultiSite_WES_Patient1	MR01_034	Female	Acral										PD21941d	Tumour	09/2014	Distant lymph node	Left groin node	39.3
MultiSite_WES_Patient1	MR01_034	Female	Acral										PD21941e	Tumour	04/2015	Distant skin/subcutanoues	Right shoulder deposit	53.7
MultiSite_WES_Patient1	MR01_034	Female	Acral										PD21941h	Tumour	04/2015	Distant lymph node	Right axilla	61.8
MultiSite_WES_Patient1	MR01_034	Female	Acral										PD21941a	Tumour	03/2014	Distant skin/subcutanoues	Left Lower limb	67.8
MultiSite_WES_Patient1	MR01_034	Female	Acral										PD31210b	Normal	NA	Normal	Normal	107.5
MultiSite_WES_Patient2	MR01_014	Male	Cutaenous	07/2012	T1b			11/2014	0	None			PD13325b	Normal	11/2012	Normal	Normal	63.2
MultiSite_WES_Patient2	MR01_014	Male	Cutaenous										PD13325a	Tumour	11/2012	Primary	Right forearm	54
MultiSite_WES_Patient2	MR01_014	Male	Cutaenous										PD13325c	Tumour	08/2014	Distant skin/subcutanoues	Right head	94.7
MultiSite_WES_Patient3	MR01_020	Male	Cutaenous	07/2012	T4b	N0	M0	03/2013	3	Vemurafenib	03/2013	05/2013	PD15682b	Normal	NA	Normal	Normal	74.2
MultiSite_WES_Patient3	MR01_020	Male	Cutaenous							Ipilimumab	05/2013	'08/2013	PD15682c	Tumour	05/2013	Regional lymph node	Axillary lymph node	64.8
MultiSite_WES_Patient3	MR01_020	Male	Cutaenous							Dacarbazine, Cisplatin, Vinblastine	11/2008	12/2009	PD15682a	Tumour	03/2013	Regional lymph node	Left axilla lymph node	68.7
MultiSite_WES_Patient4	MR01_003	Male	Cutaenous	03/2004	T4a	N1b	M1a	02/2012	4	Dacarbazine	04/2004	07/2004	PD13315b	Normal	NA	Normal	Normal	71.9
MultiSite_WES_Patient4	MR01_003	Male	Cutaenous							Ipilimumab	09/2011	11/2011	PD13315c	Tumour	02/2012	Distant skin/subcutanoues	Left back	42.2
MultiSite_WES_Patient4	MR01_003	Male	Cutaenous							Vemurafenib	02/2012	11/2013	PD13315a	Tumour	02/2012	Distant skin/subcutanoues	Right chest	44.5
MultiSite_WES_Patient4	MR01_003	Male	Cutaenous							Nivolumab	12/2013	05/2016						
MultiSite_WES_Patient5	MR01_010	Male	Cutaenous	10/2004	T2b	N0	M0	07/2012	3	Post-op RT	04/2010	04/2010	PD13615a2	Tumour	07/2012	Regional skin/subcutaneous	Skin unspecified	33.3
MultiSite_WES_Patient5	MR01_010	Male	Cutaenous							Vemurafenib	07/2012	01/2013	PD13615b	Normal	NA	Normal	Normal	35.1
MultiSite_WES_Patient5	MR01_010	Male	Cutaenous							Ipilimumab	01/2013	02/2013	PD13615d	Tumour	01/2013	Regional skin/subcutaneous	Left lower back	48.9
MultiSite_WES_Patient6	MR01_012	Male	Cutaenous	01/2010	TX	N0		11/2012	1	Dabrafenib + trametinib	10/2012	06/2013	PD13323d	Tumour	10/2012	Distant lymph node	Left groin node	46
MultiSite_WES_Patient6	MR01_012	Male	Cutaenous										PD13323b	Normal	NA	Normal	Normal	79.8
MultiSite_WES_Patient6	MR01_012	Male	Cutaenous										PD13323e	Tumour	04/2013	Distant lymph node	Left inguinal node	51.3
MultiSite_WES_Patient7	MR01_004	Male	Cutaenous	10/2010	T3b	N3	M0	04/2012	5	RT (40Gy/20#)	05/2011	06/2011	PD13316b	Normal	NA	Normal	Normal	74.9
MultiSite_WES_Patient7	MR01_004	Male	Cutaenous							Vemurafenib	04/2012	01/2013	PD13316a	Tumour	04/2012	Distant skin/subcutanoues	Left posterior ear	42.4
MultiSite_WES_Patient7	MR01_004	Male	Cutaenous							Ipilimumab	01/2013	03/2013	PD13316d	Tumour	05/2013	Distant skin/subcutanoues	Left supraclavicular fossa	52.2
MultiSite_WES_Patient7	MR01_004	Male	Cutaenous							Pazopanib	07/2013	09/2013	PD13316e	Tumour	11/2013	Regional skin/subcutaneous	Left scalp	63.6
MultiSite_WES_Patient7	MR01_004	Male	Cutaenous							Dabrafenib + trametinib	11/2013	02/2014						

Supplementary tables key

**Header**	**Code**	**Explanation**
Patient	Patient	Patient identifier as labelled in this study
MelResist_PatientID	MelResist_PatientID	Patient code within the clinical trial (Melresist)
Sex	Male	Male
Sex	Female	Female
Melanoma subtype	Cutaneous	Cutaneous/acral primary melanoma subtype
Melanoma subtype	Acral	
Date of initial melanoma diagnosis	Date of initial melanoma diagnosis	Date of initial melanoma diagnosis
TSTAGE (at initial melanoma diagnosis)	TX	Primary tumour cannot be assessed
TSTAGE (at initial melanoma diagnosis)	T0	No evidence of primary tumour
TSTAGE (at initial melanoma diagnosis)	Tis	Melanoma in situ
TSTAGE (at initial melanoma diagnosis)	T1a	Melanomas 1.0mm or less without ulceration & mitosis
TSTAGE (at initial melanoma diagnosis)	T1b	Melanomas 1.0mm or less with ulceration & mitosis
TSTAGE (at initial melanoma diagnosis)	T2a	Melanomas 1.01 - 2.0mm without ulceration
TSTAGE (at initial melanoma diagnosis)	T2b	Melanomas 1.01 - 2.0mm with ulceration
TSTAGE (at initial melanoma diagnosis)	T3a	Melanomas 2.01 - 4.0mm without ulceration
TSTAGE (at initial melanoma diagnosis)	T3b	Melanomas 2.01 - 4.0mm with ulceration
TSTAGE (at initial melanoma diagnosis)	T4a	Melanomas more than 4.0mm without ulceration
TSTAGE (at initial melanoma diagnosis)	T4b	Melanomas more than 4.0mm with ulceration
NSTAGE (at initial melanoma diagnosis)	NX	Patients in whom the regional lymph nodes cannot be assessed
NSTAGE (at initial melanoma diagnosis)	N0	No regional lymph node metastasis detected
NSTAGE (at initial melanoma diagnosis)	N1a	Melanoma cells in one lymph node with micrometastasis
NSTAGE (at initial melanoma diagnosis)	N1b	Melanoma cells in one lymph node with macrometastasis
NSTAGE (at initial melanoma diagnosis)	N2a	Melanoma cells in 2 or 3 lymph nodes with micrometastasis
NSTAGE (at initial melanoma diagnosis)	N2b	Melanoma cells in 2 or 3 lymph nodes with macrometastasis
NSTAGE (at initial melanoma diagnosis)	N2c	Melanoma cells in 2 or 3 lymph nodes with intransit met(s)/ satellite(s) without metastatic nodes
NSTAGE (at initial melanoma diagnosis)	N3	Four or more metastatic lymph nodes, or intransit met(s)/ satellite(s) with metastatic nodes
MSTAGE (at initial melanoma diagnosis)	M0	No detectable evidence of distant metastasis
MSTAGE (at initial melanoma diagnosis)	M1a	Metastasis to skin, subcutaneous, or distant lymph nodes with normal LDH
MSTAGE (at initial melanoma diagnosis)	M1b	Metastasis to lung with normal LDH
MSTAGE (at initial melanoma diagnosis)	M1c	Metastasis to all other visceral sites with normal LDH or Any distant metastasis to any site with elevated LDH
Date of trial registration	Date of trial registration	Date of trial registration
Number of lines of therapy	Number of lines of therapy	Number of lines of therapy (up to data extract 15.11.19)
Treatment name	Treatment name	Treatment name
Treatment start date	Treatment start date	Treatment start date
Treatment end date	Treatment end date	Treatment end date
WGS_SampleID	WGS_SampleID	Sample IDs for whole-genome sequencing data (index autopsy case only, though the two samples from primary tumour were sequenced with custom capture pull-down)
WES_SampleID	WES_SampleID	Sample IDs for whole-exome sequencing data
AffySampleID	AffySampleID	Affymetrix sample IDs (index autopsy case only)
Tumour/Normal	Tumour	Sample is a tumour
Tumour/Normal	Normal	Sample is normal (germline)
Sample_resection_date	Sample_resection_date	Date tumour sample was resected from patient
Sample_type	Sample_type	Type of sample
Site	Site	Anatomical site of sample
Depth	Depth	Average depth across the entire whole genome (index autopsy case only)
Purity_Estimate	Purity_Estimate	Estimate of tumour purity from copy number call (Battenberg, index autopsy case only)
Target_Region_Coverage_AfterPCRDupRemoval	Target_Region_Coverage_AfterPCRDupRemoval	Depth after excluding PCR dups, QC failed reads, supplementary and secondary read alignments
